# Temporal clustering of neuroblastic tumours in children and young adults from Ontario, Canada

**DOI:** 10.1186/s12940-022-00846-y

**Published:** 2022-03-07

**Authors:** Louise Hayes, Nermine Basta, Colin R. Muirhead, Jason D. Pole, Paul Gibson, Bruna Di Monte, Meredith S. Irwin, Mark Greenberg, Deborah A. Tweddle, Richard J. Q. McNally

**Affiliations:** 1 Population Health Sciences Institute & Newcastle University Centre for Cancer, Newcastle University, Sir James Spence Institute, Royal Victoria Infirmary, Queen Victoria Road, Newcastle upon Tyne, NE1 4LP UK; 2 Pediatric Oncology Group of Ontario, Toronto, Canada; 3 Centre for Health Services Research, The University of Queensland, Brisbane, Australia; 4 Division of Paediatric Hematology/Oncology, McMaster University, Hamilton, Canada; 5 Department of Paediatrics, The Hospital for Sick Children, Toronto, Canada; 6 Wolfson Childhood Cancer Research Centre, Translational and Clinical Research Institute, Newcastle University Centre for Cancer, Newcastle University, Newcastle upon Tyne, UK; 7 Great North Children’s Hospital, Newcastle upon Tyne, UK; 8 Newcastle University Centre for Cancer, Newcastle University, Sir James Spence Institute, Royal Victoria Infirmary, Queen Victoria Road, Newcastle upon Tyne, NE1 4LP UK

**Keywords:** Epidemiology, Neuroblastic tumours, Temporal clustering

## Abstract

**Background:**

The aetiology of neuroblastic tumours is likely to involve both genetic and environmental factors. A number of possible environmental risk factors have been suggested, including infection. If an irregular temporal pattern in incidence is found, this might suggest that a transient agent, such as an infection, is implicated. Previous work has found evidence for temporal clustering in children and young adults living in northern England.

**Methods:**

We examined data from a second population-based registry from Ontario, Canada to determine whether there was evidence of temporal clustering of neuroblastic tumours. Cases diagnosed in children and young adults aged 0-19 years between 1985 and 2016 were extracted from the population-based Pediatric Oncology Group of Ontario Networked Information System (POGONIS). A modified version of the Potthoff-Whittinghill method was used to test for temporal clustering. Estimates of extra-Poisson variation (EPV) and standard errors (SE) were obtained.

**Results:**

Eight hundred seventy-six cases of neuroblastic tumours were diagnosed during the study period. Overall, no evidence of temporal clustering was found between fortnights, between months or between quarters within years. However, significant EPV was found between years within the full study period (EPV = 1.05, SE = 0.25; *P* = 0.005).

**Conclusions:**

The findings are consistent with the possibility that a transient agent, such as an infection that is characterised by ‘peaks and troughs’ in its occurrence, might be implicated in the aetiology of neuroblastic tumours. However, this pattern may also reflect a long-term increase in the numbers of cases, rather than peaks and troughs.

## Introduction

Approximately 60-90 cases of neuroblastic tumour are diagnosed annually in children and young adults in Canada [1], and they are the most common cancer diagnosed in children below the age of 12 months, accounting for approximately 30% of all cancers diagnosed in infants. The incidence of neuroblastic tumours has remained relatively stable over recent decades [2, 3]. Although aetiology is poorly understood, a number of studies have indicated a role for both genetic and environmental factors. The quality of the evidence regarding specific environmental factors is variable. However, epidemiological studies that have suggested associations with increased risk include higher number of siblings, pesticides, ambient air toxic exposures during pregnancy, maternal use of some medications, alcohol consumption and smoking during pregnancy. Protective effects were associated with infections in childhood, breast-feeding, vitamin supplementation, maternal fetal loss, folic acid, allergies and Down syndrome (studies reviewed by Muirhead and colleagues [4]).

‘Temporal clustering’ is a general non-regular temporal distribution of cases that is not restricted to one particular time period. This type of clustering might occur when there a smaller number of relatively long time periods (of the order of years or several months) with markedly increased incidence, or a larger number of relatively short time periods (of the order of weeks or a month or so) with moderately increased incidence. This irregular occurrence contrasts with seasonal variation that occurs each year at similar times. Different statistical methods are used to detect such seasonal variation. The method used here is based on a test originally developed by Potthoff and Whittinghill to detect extra-Poisson variation (EPV) [5, 6].

A previous study from northern England found statistically significant temporal clustering amongst 227 cases of neuroblastic tumour who were diagnosed during the period 1968-2011 at ages 0-24 years. The findings from northern England were interpreted as providing support for the role of a transient environmental agent in aetiology. Such an agent would be expected to have widespread occurrence and would display as ‘mini-epidemics’ [4].

The present study aimed to explore temporal clustering of neuroblastic tumours in a much larger population of children and young adults (aged 0 - 19 years) from Ontario, Canada.

## Methods

All cases aged 0-19 years, diagnosed with a neuroblastic tumour (neuroblastoma or ganglioneuroblastoma) during the period 1985-2016 were extracted from the Paediatric Oncology Group of Ontario Networked Information System (POGONIS). This registry started in 1985 and includes cases of all malignancies in children and young people aged 0-19 years, diagnosed and treated at five centres throughout Ontario (Children’s Hospital of Eastern Ontario, Ottawa; Children’s Hospital, London Health Sciences Centre, London; Kingston General Hospital, Kingston; McMaster Children’s Hospital, Hamilton; and The Hospital for Sick Children, Toronto). Comparison with the Ontario Cancer Registry showed that completeness of ascertainment was 96-98% for children aged 0-14 years, but for those aged 15-19 years the estimate of ascertainment was 50% because some patients were referred to adult clinics [7–12]. There were only 5 cases of neuroblastic tumours aged 15-19 years included in the present study analyses. The population of the study region aged 0-19 years is approximately 3 million [13].

### Ethics approval

The study has ethical approval from the Western University Health Science Research Ethics Board (initial approval date 14th June 2017).

### Prior hypothesis

The aetiological hypothesis tested was that a primary factor affecting temporal variation in the diagnosis of neuroblastic tumours is related to exposure to a spatially widespread, non-regular environmental agent which varies in intensity with time and which occurs close to the date of diagnosis or at very similar times preceding diagnosis. Examples of putative agents include infections and air pollution. Timely diagnosis of neuroblastic tumours presents challenges [14]. However, one study has found that the median lag time between symptom onset and diagnosis of neuroblastic tumours was 21 days [15].

### Statistical analysis

The methodology that was applied has been used in previous analyses of temporal clustering [4, 16, 17]. The approach applied involved an adapted version [16] of a method that was originally developed by Potthoff and Whittinghill [5, 6]. This method was used to determine the amount of EPV in the numbers of diagnoses of neuroblastic tumour per fortnight, calendar month, quarter of a year and calendar year. An assumption was made that the numbers of diagnoses followed a negative binomial distribution in which the ratio of the variance to the expected number of diagnoses was equal to a constant, denoted as 1+ β. When β is equal to zero, then the distribution of the numbers of diagnoses is Poisson. However, if β is greater than zero, then the number of diagnoses demonstrates EPV. They are said to be over-dispersed compared with the Poisson distribution. Estimation of EPV, together with its standard error, was based on the score statistic [18]. Tests for EPV were one-sided (β > 0). *P* values were determined using 10,000 simulations under the assumption that β = 0. Statistical significance was assessed using a critical value of *P* < 0.05. The code for this modified method is available from the authors on request.

When analysing short-term patterns, the role of longer-term variability was removed by conditioning on the total number of cases within a calendar month, quarter of a year (i.e. January to March; April to June; July to September; October to December), calendar year or the complete length of the study period. There was a focus on analyses of: (i) ‘between fortnights within months’, (ii) ‘between months within quarters’, (iii) ‘between quarters within years’ and (iv) ‘between years’. The interpretation of each of these analyses is independent of the other analyses because they are conditional.

Date of diagnosis is defined as the date of pathological confirmation. Date of diagnosis was always known. The definition of fortnights has been described previously [16]. Pragmatically, they were taken to be the first 15 days of the calendar month (or the first 14 days for February), compared to the remainder of the month. The calculation of expected number of cases within a given period took account of variations in the lengths of fortnights, months and years.

The expected number of cases was assumed to be proportional to the length of the relevant period. Standardisation of the expected numbers was based on the assumption that the totals were equal to the observed totals for the same period. Adjustments for ‘within years’ analyses were not needed because changes in population size within years was assumed to be negligible. For ‘between years’ analyses, the analyses reported are not adjusted for population size. However, sensitivity analyses with adjustment for population size for all cases (0-19 years) and for those aged < 18 months were performed. It should be noted that data on the population aged 0-18 months were not available so we estimated it as 1.5 times the 0-12 month population.

The earlier study from northern England had identified specific differences between the nature of the temporal clustering at age < 18 months and at older ages during childhood and young adulthood. That study also identified differences between males and females [4]. In order to test the hypotheses that differences might pertain in the type of temporal clustering, analyses were also conducted at age < 18 months and at age 18 months - 19 years, and, separately, for males and females for all ages 0-19 years.

## Results

**Fig. 1:**
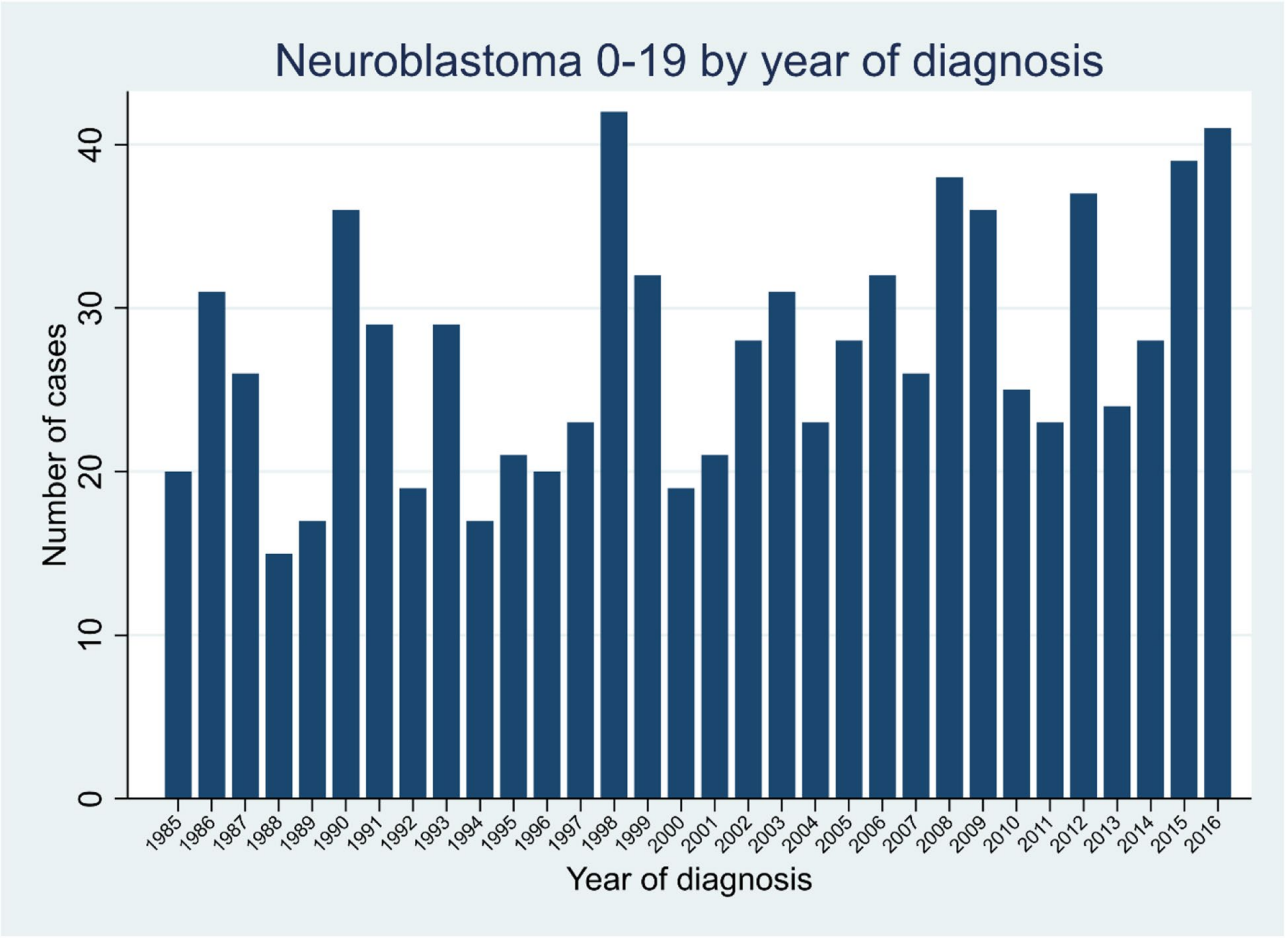
Number of cases per year of neuroblastic tumours at ages 0-19 years in Ontario, Canada. The number of cases by calendar year of diagnosis are shown

A total of 876 cases aged 0-19 years were diagnosed in Ontario, Canada during the period 1985-2016. Table 1 presents the number of cases categorised by sex, age at diagnosis and time period of diagnosis. The number of cases by year of diagnosis are displayed in Fig. 1. Overall, there was evidence for long-term patterns of occurrence, as demonstrated by the significant EPV that was found between years and between quarters of years, with EPV equal to 1.05 (SE = 0.25, *P* = 0.005) for the analysis between years and EPV equal to 0.41 (SE = 0.13, *P* = 0.002) for the analysis between quarters within the full study period. Conversely, the Potthoff-Whittinghill analyses between fortnights, between months and between quarters within years did not find any evidence for extraneous variability (Table 2). Adjustment for long-term variation in population size had little impact on the analysis between years.

**Table 1:** Characteristics of cases of neuroblastic tumours in the study population (based on cases diagnosed during 1985-2016 inclusive)

		Number of cases (%)	
Overall (0-19 years)		876 (100)	
Males		481 (54.9)	
Females		395 (45.1)	
Age at diagnosis:			
< 18 months		393 (44.9)	
18 months – 4 years		364 (41.6)	
5 – 19 years		119 (13.6)	
Time period:			
1985-1992		193 (22.0)	
1993-2000		203 (22.2)	
2001-2008		227 (25.9)	
2009-2016		253 (28.9)	
	Mean	Median	Quartile 1, Quartile 3
Age at diagnosis (years)	2.57	1.77	0.67, 3.66
Annual number of cases	27.4	27	21, 32

**Table 2:** Analyses of temporal clustering of neuroblastic tumours at ages 0-19 years

Type of analysis	^β^1^ (SE)			
	*One-sided p value*			
	Within months	Within quarters	Within years	Within full period
Between fortnights	−0.07 (0.11) *P = 0.714*	− 0.11 (0.06) *P = 0.973*	− 0.06 (0.05) *P = 0.881*	− 0.01 (0.05) *P = 0.535*
Between months		− 0.13 (0.10) *P = 0.910*	− 0.07 (0.08) *P = 0.812*	0.04 (0.07) *P = 0.259*
Between quarters			0.14 (0.15) *P = 0.17*	**0.41 (0.13)** ***P = 0.002***
Between years				**1.05 (0.25)** ***P = 0.005***

^1^ ^β is the one-step estimate of β, the extra-Poisson variation, calculated in the same way as originally described by Muirhead [16]

A separate analysis of cases aged < 18 months demonstrated that EPV was only apparent between quarters, with EPV equal to 0.37 (SE = 0.15, *P* = 0.014) for the analysis between quarters within years, and EPV equal to 0.34 (SE = 0.13, *P* = 0.007) for the analysis between quarters within the full study period. However, there was no evidence for extraneous variability between fortnights and between months (Table 3). The sensitivity analysis found that adjusting for population size did not affect the findings at < 18 months.

**Table 3:** Analyses of temporal clustering of neuroblastic tumours at ages < 18 months

Type of analysis	^β^1^ (SE)			
	*One-sided p value*			
	Within months	Within quarters	Within years	Within full period
Between fortnights	−0.271 (0.19) *p = 0.926*	− 0.103 (0.08) *p = 0.910*	− 0.019 (0.05) *p = 0.632*	*−*0.006 (0.05) *p = 0.537*
Between months		−0.05 (0.12) *p = 0.656*	0.09 (0.08) *p = 0.144*	0.08 (0.07) *p = 0.141*
Between quarters			**0.37 (0.15)** ***p = 0.014***	**0.34 (0.13)** ***p = 0.007***
Between years				0.35 (0.27) *p = 0.106*

^1^ ^β is the one-step estimate of β, the extra-Poisson variation, calculated in the same way as originally described by Muirhead [16]

In contrast, for cases aged 18 months to 19 years the analyses found that EPV was only present between years, with EPV equal to 0.64 (SE = 0.27, *P* = 0.018) for the analysis between years within the full study period. There was no evidence for extraneous variation between fortnights, between months, or between quarters (Table 4).

**Table 4:** Analyses of temporal clustering of neuroblastic tumours at ages 18 months to 19 years

Type of analysis	^β^1^ (SE)			
	*One-sided p value*			
	Within months	Within quarters	Within years	Within full period
Between fortnights	−0.11 (0.17) *p = 0.738*	− 0.14 (0.07) *p = 0.979*	− 0.08 (0.95) *p = 0.933*	−0.06 (0.05) *p = 0.867*
Between months		−0.20 (0.11) *p = 0.968*	−0.11 (0.08) *p = 0.926*	− 0.07 (0.07) *p = 0.827*
Between quarters			0.02 (0.15) *p = 0.443*	0.18 (0.13) *p = 0.064*
Between years				**0.64 (0.27)** ***p = 0.018***

^1^ ^β is the one-step estimate of β, the extra-Poisson variation, calculated in the same way as originally described by Muirhead [16]

Males and females were analysed separately. There was no evidence of EPV amongst male cases (Table 5). For females, there was evidence for EPV between years within the full study period, with EPV equal to 0.79 (SE = 0.25, *P* = 0.005), as well as between quarters within the full study period, with EPV equal to 0.31 (SE = 0.13, *P* = 0.012, Table 6).

**Table 5:** Analyses of temporal clustering of neuroblastic tumours at ages 0-19 years for males

Type of analysis	^β^1^ (SE)			
	*One-sided p value*			
	Within months	Within quarters	Within years	Within full period
Between fortnights	0.18 (0.16) *p = 0.411*	−0.02 (0.07) *p = 0.610*	−0.03 (0.05) *p = 0.717*	− 0.04 (0.05) *p = 0.773*
Between months		−0.16 (0.11) *p = 0.925*	−0.15 (0.08) *p = 0.975*	− 0.15 (0.07) *p = 0.985*
Between quarters			0.17 (0.15) *p = 0.874*	−0.14 (0.13) *p = 0.859*
Between years				−0.12 (0.27) *p = 0.652*

^1^ ^β is the one-step estimate of β, the extra-Poisson variation, calculated in the same way as originally described by Muirhead [16]

**Table 6:** Analyses of temporal clustering of neuroblastic tumours at ages 0-19 years for females

Type of analysis	^β^1^ (SE)			
	*One-sided p value*			
	Within months	Within quarters	Within years	Within full period
Between fortnights	−0.04 (0.193) *p = 0.581*	−0.04 (0.787) *p = 0.672*	− 0.01 (0.06) *p = 0.538*	0.04 (0.05) *p = 0.207*
Between months		−0.12 (0.12) *p = 0.835*	−0.02 (0.08) *p = 0.579*	0.07 (0.07) *p = 0.176*
Between quarters			0.11 (0.15) *p = 0.230*	**0.31 (0.13)** ***p = 0.012***
Between years				**0.79 (0.25)** ***p = 0.005***

^1^ ^β is the one-step estimate of β, the extra-Poisson variation, calculated in the same way as originally described by Muirhead [16]

## Discussion

Our findings suggest evidence of temporal clustering of neuroblastic tumours in children and young people over more prolonged periods of time (between years). There was no evidence of EPV over shorter time periods (i.e. fortnights or months). Temporal clustering was only apparent between quarters for cases aged < 18 months, and, in contrast, only between years for cases aged 18 months to 19 years. Overall, temporal clustering was exhibited only for neuroblastic tumours in girls and not boys.

There is a paucity of literature concerning the aetiology of neuroblastic tumours. Hereditary neuroblastoma occurs in 1-2% of cases, most notably due to germline mutations in *ALK* [19–21]. One study found that aetiological risk factors related to the prenatal and perinatal period may be associated with the age of diagnosis [22]. Another study showed that congenital anomalies and pre-eclampsia were associated with increased risk of neuroblastic tumours at age < 18 months [23]. A pooled analysis of French studies demonstrated that congenital malformations and fetal growth anomalies conferred increased risk of a neuroblastic tumour [24].

To our knowledge the present study is only the second study to demonstrate temporal clustering amongst cases of neuroblastic tumours, although a specific temporal cluster was identified using different methodology in a study from the province of Cordoba, Argentina [25]. However, the findings of the present study from Canada contrast with those of a previous study from northern England [4]. The Canadian data demonstrated temporal clustering between years, whereas the data from northern England showed that clustering was apparent between fortnights or between months. In addition, the present study found that temporal clustering was only present for females and not for males. This contrasts with the findings from northern England where clustering was exhibited amongst both males and females, but was far more pronounced amongst males. The reasons for these differences between the two studies are not clear. However, we might speculate that different demographics and socioeconomic conditions might lead to markedly distinct patterns of exposure to an unknown aetiological agent or agents. There are similarities in the overall levels of deprivation between Ontario and the whole of the UK. However, there are distinct differences in the nature of deprivation between the two locations [26, 27]. The population of Ontario is approximately 14.7 million, is ethnically diverse and covers a large geographical area of 917,741 km^2^. Although the province comprises large rural parts, approximately 11.3 million live in metropolitan areas [28]. In contrast, the northern region of England has a total population of approximately 3.1 million and covers an area of 15,337 km^2^ with a mixture of urban and rural areas. Population density varies widely. Northumberland and Cumbria are the two most sparsely populated counties in England, while Tyne & Wear is one of the most densely populated. Almost 1.4 million people live in the more urban areas of Newcastle upon Tyne, Gateshead, Sunderland, Middlesbrough, North and South Tyneside. The population of northern England is predominantly Caucasian and ethnic minorities accounted for under 2% during the study period. The northern region is one of the most deprived in England [29–31].

The lengthier temporal periods seen in the present study suggest more prolonged lag times for the spread of a geographically widespread environmental agent. Alternatively, it is possible that there may have been more variability in the length of the time between development and diagnosis of a neuroblastic tumour in Ontario compared to northern England. The temporal period was shorter for the younger age group (< 18 months), where clustering occurred between quarters, than for the older age group (18 months – 19 years), where clustering occurred between years. This suggests shorter lag times for the younger age group, which is consistent with the previous study from northern England [4]. It is also possible that this pattern of occurrence might reflect a long-term increase in incidence. The differences between the studies in the findings based on gender should be interpreted with caution, as there is no readily apparent explanation. However, we might speculate that this may be related to differences in lag time from exposure to onset, or differences in patterns of exposure to putative environmental risk factors, between males and females. Gender differences in the pattern of occurrence of neuroblastic tumours have been noted previously [32]. It should be acknowledged that chance may play a role in the differences found specifically for temporal clustering between the two studies. Further research is needed to provide a clearer explanation.

The findings from both the present study and the previous study from northern England [4] provide support for the involvement of a temporally varying environmental agent. Other descriptive studies have analysed space-time clustering and spatial clustering amongst cases of neuroblastic tumours [33–38]. However, there was inconsistency between the findings from these studies. This suggests that the environmental agent or agents involved only rarely lead to the initiation of a neuroblastic tumour, or that the studies had varying sensitivity to identify the impacts of these agents.

Some methodological issues should be noted. The method used in this study is based on the idea that the form that any temporal clustering might take is unknown. Consequently, the approach taken does not base the analysis on a particular model of temporal clustering. Muirhead and Potthoff and Whittinghill have considered the power of this approach to detect EPV [5, 6, 18]. Muirhead, with reference to a comparative study organised by the International Agency for Research on Cancer, concluded that this methodology has reasonable power to detect EPV under a range of models for over-dispersion, including those for which the constant variance: incidence assumptions does not hold [39]. If there were seasonal variation that cut across quarters, then the approach used here might well detect EPV. Methods that look specifically for seasonal variation would have greater power to detect such variation. However, other studies provide little evidence of seasonal variation in neuroblastic tumours and, in any case, the focus of the current analysis is on variation that need not be seasonal [40, 41]. The time periods used are arbitrary, but, given that the analysis covers such a long period (more than 30 years), the particular choice of fortnights, months and years should not affect the ability to detect short-term and/or longer-term variation. We recognise that some of the results exhibit large uncertainties in the estimates due to the small number of events, and thus should not be over interpreted.

In conclusion, this study of neuroblastic tumours from Canada has found evidence of temporal clustering. However, the scale of the temporal clustering differs from a previous study from northern England. In contrast to the findings from that study, the Canadian data have demonstrated that the clustering was confined to more prolonged temporal intervals (principally years, rather than shorter periods). The temporal clustering found in the present study may be either characterised by ‘peaks and troughs’ or by a long-term increase in the number of cases. Both of these scenarios are consistent with the involvement of one or more widespread environmental agents in aetiology. It is possible that different environmental agents are involved in Canada and northern England, and this could explain the differences in patterns that has been seen. Further research is needed to identify putative aetiological agents. In addition, larger studies of temporal clustering could be undertaken (e.g. by the recording of date of diagnosis in the International Neuroblastoma Risk Group database, which would require information on the geographical region or country for each case).

## Data Availability

The dataset analysed during the current study are not publicly available due to the confidential nature of the data.
